# Evaluation of the Patient Acceptable Symptom State in a pooled analysis of two multicentre, randomised, double-blind, placebo-controlled studies evaluating lumiracoxib and celecoxib in patients with osteoarthritis

**DOI:** 10.1186/ar2118

**Published:** 2007-01-31

**Authors:** Maxime Dougados, Alan Moore, Shaohua Yu, Xavier Gitton

**Affiliations:** 1 Department of Rheumatology, Hôpital Cochin, 27 Rue du Faubourg Saint Jacques, 75014 Paris, France; 2 Biostatistics, Novartis Pharma AG, Lichtstrasse 35, CH-4056 Basel, Switzerland; 3 Biostatistics, Novartis Pharmaceuticals Corporation, One Health Plaza, East Hanover, NJ 07936, USA; 4 Clinical Development & Medical Affairs, Novartis Pharma AG, Lichtstrasse 35, CH-4056 Basel, Switzerland

## Abstract

Patient Acceptable Symptom State (PASS) is an absolute threshold proposed for symptomatic variables in osteoarthritis (OA) to determine the point beyond which patients consider themselves well and, as such, are satisfied with treatment. Two large previously reported studies of knee OA have shown that both lumiracoxib and celecoxib were superior to placebo in terms of conventional outcome measures. To assess the clinical relevance of these results from the patient's perspective, the same data pooled from these two studies were analysed with respect to the PASS. In total, 3,235 patients were included in two multicentre, randomised, double-blind studies of identical design. Patients were randomly assigned to receive lumiracoxib 100 mg once daily (*n *= 811), lumiracoxib 100 mg once daily with an initial dose of lumiracoxib 200 mg once daily for the first 2 weeks (100 mg once daily with initial dose [*n *= 805]), celecoxib 200 mg once daily (*n *= 813), or placebo (*n *= 806) for 13 weeks. Treatments were compared with respect to the PASS criteria (for OA pain, patient's global assessment of disease activity, and the Western Ontario and McMaster Universities Osteoarthritis Index Likert version 3.1 [WOMAC™ LK 3.1] Function [difficulty in performing daily activities] subscale score). At week 13, 43.3%, 45.3%, and 42.2% of patients in the lumiracoxib 100 mg once daily, lumiracoxib 100 mg once daily with initial dose, and the celecoxib 200 mg once daily groups, respectively, considered their current states as satisfactory versus 35.5% in the placebo group. Similar results were observed for patient's global assessment of disease activity and WOMAC™ LK 3.1 Function subscale score. This post hoc analysis suggests that the statistical significance of the results observed with lumiracoxib or celecoxib compared with placebo using conventional outcome variables is complemented by clinical relevance to the patient. Trial registration numbers: NCT00366938 and NCT00367315.

## Introduction

In 2003, the Outcome Measures in Clinical Trials (OMERACT) 6 meeting emphasised the importance of defining clinical trial outcomes that are comprehensive and can be used to influence clinical decision-making [1]. The question for many clinicians is whether changes in self-reported levels of pain on a 0- to 100-mm visual-analogue scale (VAS) are clinically important and whether they reflect a meaningful improvement for the patient. Clinicians strongly favour the presentation of results at an individual level rather than a group level (as expressed by the mean change in symptom score) [2]. The challenge of the meeting was to determine the minimal meaningful change in a score for an individual by means of a structured instrument.

Two concepts that reflect a meaningful clinical response from the patient's perspective have recently been developed and tested for clinical trials. These two concept measures are the Minimal Clinically Important Improvement (MCII), defined as the smallest change in a measurement which signifies an important improvement in a patient's symptom score [3], and the Patient Acceptable Symptom State (PASS), defined as the symptom score beyond which patients consider themselves to be well [2,4,5]. These measures are complementary, describing, from the patient's perspective, the concept of well-being or remission of symptoms: that is, 'feeling good' (encompassed in PASS) and the concept of improvement or 'feeling better' (encompassed in MCII) [2].

PASS provides clinically meaningful information that can be expressed as a percentage of patients who meet the threshold for PASS regardless of the change from baseline in symptoms. PASS thresholds (on a 0- to 100-mm VAS) have recently been proposed for patients with osteoarthritis (OA) of the knee. These were less than or equal to 32.3 mm for pain intensity, less than or equal to 32.0 mm for patient's global assessment of disease activity, and a score of less than or equal to 31.0 for Western Ontario and McMaster Universities Osteoarthritis Index (WOMAC™) Function (difficulty in performing daily activities) subscale score [5]. The VAS version of the WOMAC™ Function subscale must be converted to a 0- to 68-point scale if the Likert version 3.1 (LK 3.1) of the WOMAC™ questionnaire is used: the PASS threshold of less than or equal to 31.0 then converts into a threshold of less than or equal to 21.08, which must be achieved for a patient to be satisfied according to PASS. Assessment of patient satisfaction by means of the PASS criteria can be approached in a number of ways: satisfaction at the end of a study period, time taken to achieve patient satisfaction, or time taken to achieve sustained satisfaction. Time taken to achieve patient satisfaction provides an evaluation not only of the concept of 'feeling good' but also of 'feeling good as soon as possible.' Time taken to achieve sustained satisfaction combines the concept of 'feeling good as soon as possible' with the general definition of 'good condition,' thus providing a measurement of sustained good condition; it is defined as the first visit during which the value of the variable exceeds the PASS criteria and remains so until study end. In studies that incorporate a number of scheduled visits before the final one, for both time taken to achieve satisfaction and time taken to achieve sustained satisfaction, the Kaplan-Meier method can be used and the results presented using a Kaplan-Meier analysis.

In parallel to the development of the PASS criteria, MCII thresholds for absolute change in pain intensity, patient's global assessment of disease activity, and WOMAC™ Function subscale score in patients with OA of the knee (defined as the 75th percentile of the change in score among patients whose evaluation of response to treatment was 'good') were reported as -19.9, -18.3, and -9.1 mm, respectively [3]. As for PASS, the VAS version of the WOMAC™ Function subscale must be converted to a 0- to 68-point scale if the LK 3.1 version of the WOMAC™ questionnaire is used; the -9.1-mm MCII threshold then converts into a -6.19 threshold, which must be achieved for a patient to be satisfied according to MCII. The PASS and MCII thresholds have subsequently been used to facilitate the presentation and interpretation of results obtained in clinical trials [4].

Lumiracoxib is a novel, selective cyclo-oxygenase-2 (COX-2) inhibitor for the treatment of OA and acute pain. In two 13-week, international, multicentre, double-blind studies in patients with OA of the knee, it was compared with celecoxib 200 mg once daily (od) as a positive control and placebo as a negative control. Both lumiracoxib 100 mg od and celecoxib 200 mg od demonstrated a statistically significant improvement in OA symptoms compared with placebo when analysing conventional outcome variables (OA pain, patient's global assessment of disease activity, and the WOMAC™ questionnaire total score) [6,7].

Here, we report on a pooled analysis of the above two studies. This analysis evaluated the effectiveness of lumiracoxib 100 m god (with and without a 200-mg od initial dose for the first 2 weeks) and celecoxib 200 mg od compared with placebo according to the percentage of patients achieving PASS or MCII thresholds for OA pain, patient's global assessment of disease activity, and functional impairment. We present data on patient satisfaction according to PASS during and at the end of the study period and on patient achievement of sustained satisfaction by PASS.

## Materials and methods

A pooled analysis of data taken from two international, multicentre, double-blind, double-dummy, placebo-controlled, parallel-group, 13-week studies of patients with OA of the knee and of identical design was conducted. Methodology for the studies has previously been described elsewhere in detail [6,7] and both studies were registered by Clinical Trials [8] (registration numbers NCT00366938 and NCT00367315).

### Assessments and variables

The co-primary efficacy variables were OA pain intensity in the target knee, patient's global assessment of disease activity, and functional status (WOMAC™ LK 3.1 subscale) measured at study end [6,7]. Post-baseline clinic visits were at weeks 2, 4, 8, and 13 [6,7]. At any visit during the study, achievement of symptom satisfaction for OA pain, patient's global assessment of disease activity, and WOMAC™ LK 3.1 Function score was assessed by the percentage of patients achieving PASS.

### Statistical analysis

Unless otherwise stated, evaluations were performed on an intention-to-treat basis, which included all patients who had been randomly assigned to treatment and exposed to study medication. In the event of missing data, the last-observation-carried-forward technique was used. For dichotomous variables, the number needed to treat (NNT) (that is, the number of patients needed to be treated with the active treatment rather than placebo for one additional patient to benefit) was derived from the difference in response rates between active treatment and placebo.

By means of a conventional approach, the treatment effect (that is, each active treatment group versus placebo) for the three symptomatic outcome variables – OA pain, patient's global assessment of disease activity, and WOMAC™ LK 3.1 Function – was originally estimated using the least square means obtained from an analysis of covariance with study and baseline values as covariates.

The three variables were transformed to dichotomous variables (yes/no) with regard to the MCII and PASS criteria. The percentage of patients achieving improvement according to MCII was assessed at weeks 2, 4, 8, and 13 for OA pain intensity in the target knee (change greater than or equal to 19.9 mm on VAS) and patient's global assessment of disease activity (change greater than or equal to 18.3 mm on VAS) and at weeks 2, 8, and 13 for WOMAC™ LK 3.1 Function subscale score (change greater than or equal to 6.19 [converted from VAS]) [3]. The percentage of patients achieving symptom satisfaction according to PASS was assessed at weeks 2, 4, 8, and 13 for OA pain intensity in the target knee (less than or equal to 32.3 mm on VAS) and patient's global assessment of disease activity (less than or equal to 32.0 mm on VAS) and at weeks 2, 8, and 13 for the WOMAC™ LK 3.1 Function subscale score (less than or equal to 21.08 [converted from VAS]) [5]. For these variables, the treatment effect was evaluated by fitting a multiple logistic regression model with treatment as the main effect. Pairwise comparisons between treatment effects were based on the likelihood ratio tests from type III analyses. Odds ratios for the between-treatment comparisons were also presented.

For each PASS variable, the Kaplan-Meier method was used to estimate the time to achievement of first sustained PASS, defined by the first time (first visit) that the PASS threshold (32.3 mm for pain, 32.0 mm for patient's global assessment of disease activity, and 21.08 for WOMAC™ LK 3.1 Function) was exceeded and subsequently maintained during consecutive visits until week 13 (inclusive). Missing data were not imputed. Only patients with consecutive visits up to week 13 and with no values of the variable missing or above the PASS threshold at week 13 were considered to have achieved a sustained PASS; otherwise, patients were considered censored at the last date on treatment. The results are presented using Kaplan-Meier curves, and the pairwise comparisons between treatment effects were evaluated using Wilcoxon tests.

The percentage of patients achieving the threshold for at least one, two, or three PASS criteria (OA pain, patient's global assessment of disease activity, or WOMAC™ LK 3.1 Function subscale score) was calculated at weeks 2 and 13.

Finally, response to treatment according to the OMERACT-Osteoarthritis Research Society International (OMERACT-OARSI) criteria was assessed at weeks 2, 8, and 13 [6,7]. A logistic regression model was used to analyse responses to treatment according to OMERACT-OARSI criteria.

## Results

**Figure 1:**
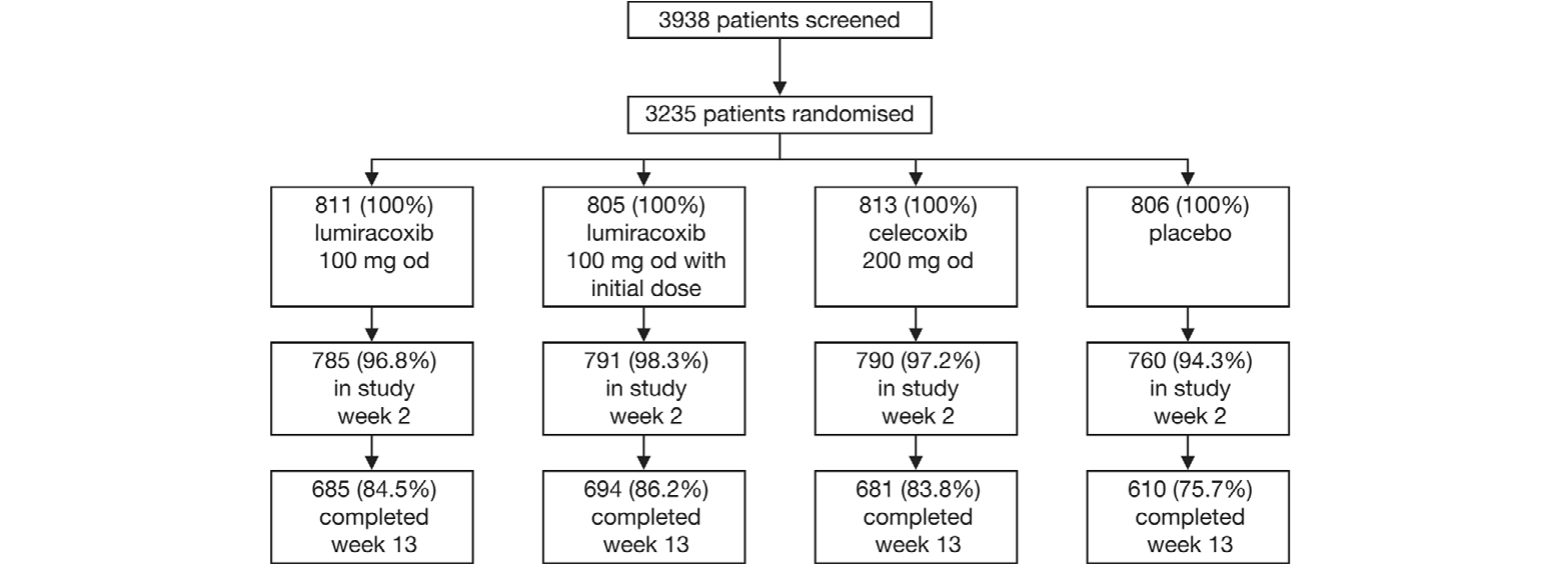
Patient flow diagram.

Patient demographics and baseline disease characteristics of the 3,235 patients included in the pooled analysis are shown in Table 1. A patient flow diagram is shown in Figure 1.

### Evaluation of each outcome variable according to different techniques

#### Final visit of the study

**Figure 2:**
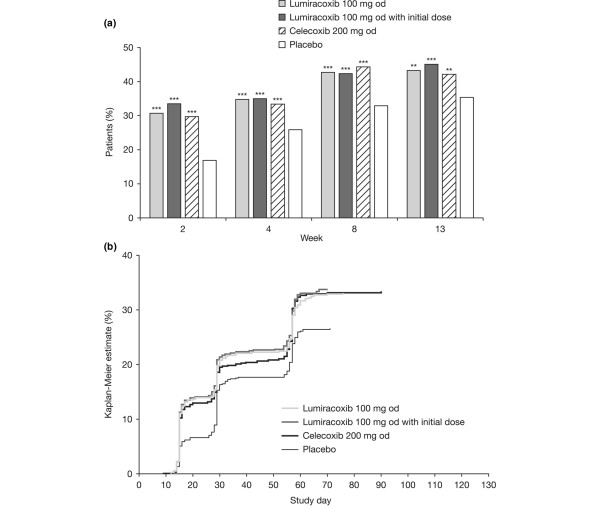
Osteoarthritis pain intensity in the target knee. **(a) **Percentage of patients considering their state as satisfactory according to Patient Acceptable Symptom State (PASS) at weeks 2, 4, 8, and 13. **P < 0.01; ***P < 0.001 versus placebo logistic regression model adjusted for multiple comparisons. **(b) **Kaplan-Meier estimate of the probability of first sustained satisfaction with treatment maintained until week 13 according to PASS. *P *< 0.001 versus placebo for all active treatments using both log-rank and Wilcoxon tests except for lumiracoxib with initial dose versus placebo using Wilcoxon (*P *< 0.0001).

**Figure 3:**
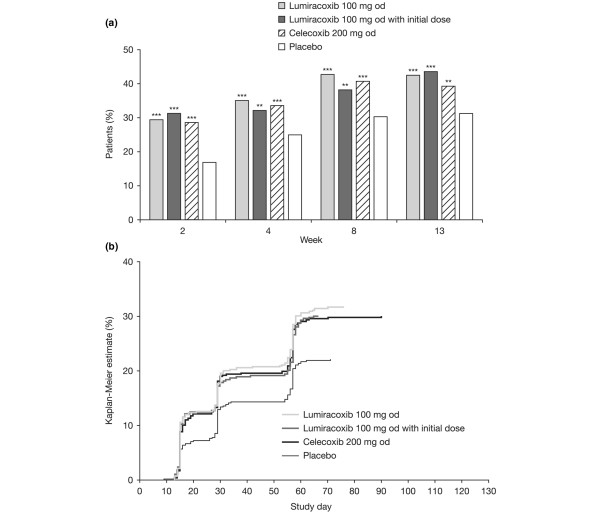
Patient's global assessment of disease activity. **(a) **Percentage of patients considering their state as satisfactory according to Patient Acceptable Symptom State (PASS) at weeks 2, 4, 8, and 13. **P < 0.01; ***P < 0.001 versus placebo logistic regression model adjusted for multiple comparisons. **(b) **Kaplan-Meier estimate of the probability of first sustained satisfaction with treatment maintained until week 13 according to PASS. *P *< 0.001 versus placebo for all active treatments using both log-rank and Wilcoxon tests.

**Figure 4:**
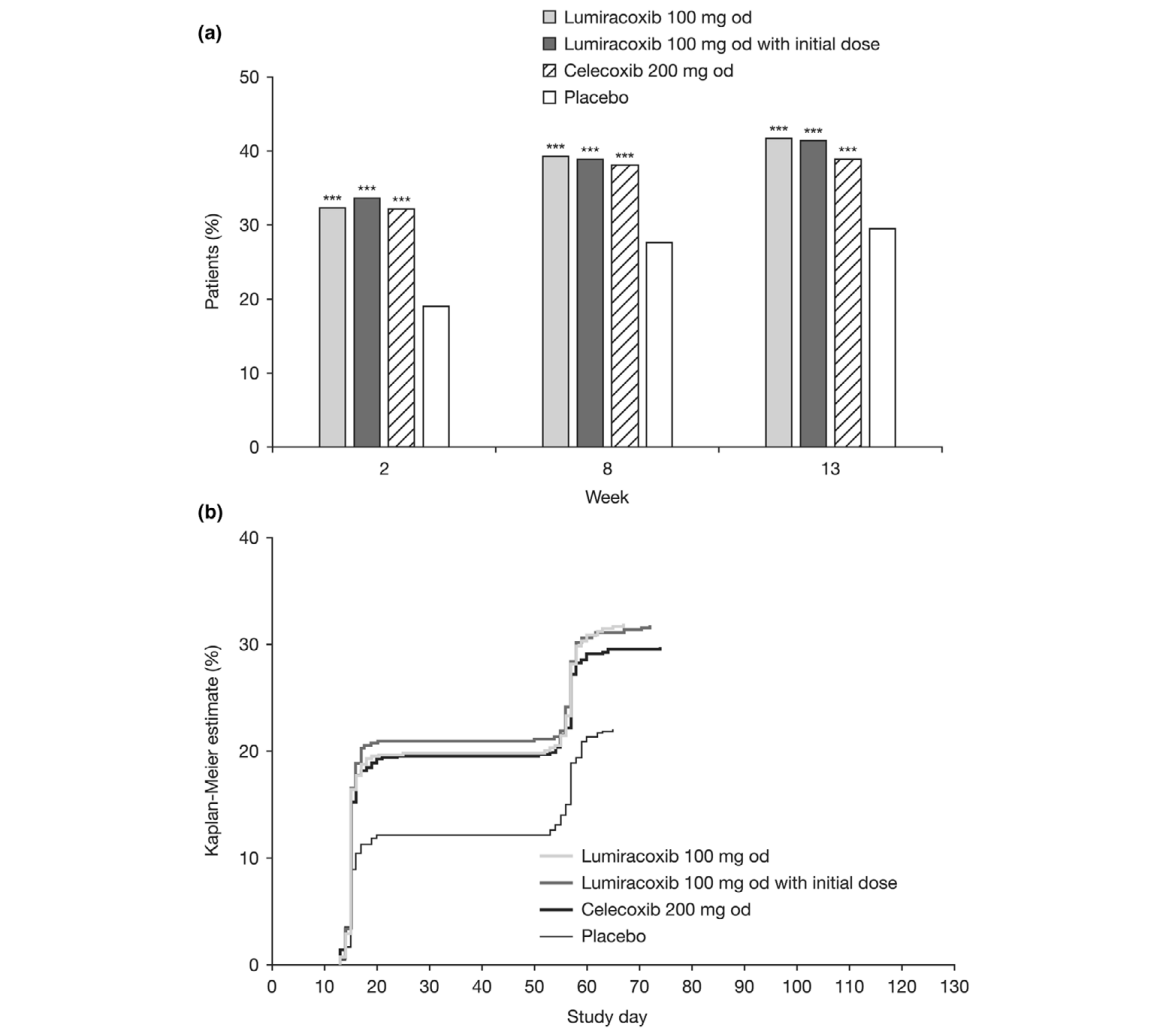
Western Ontario and McMaster Universities Osteoarthritis Index Likert version 3.1. Function subscale score. **(a) **Percentage of patients considering their state as satisfactory according to Patient Acceptable Symptom State (PASS) at weeks 2, 8, and 13. ***P < 0.001 versus placebo logistic regression model unadjusted for multiple comparisons.**(b) **Kaplan-Meier estimate of the probability of first sustained satisfaction with treatment maintained until week 13 according to PASS. *P *< 0.001 versus placebo for all active treatments using both log-rank and Wilcoxon tests.

Tables 2 to 4 summarise mean changes from baseline, patients achieving the MCII threshold, and patients achieving the PASS threshold for OA pain (Table 2; Figure 2a), the patient's global assessment of disease activity (Table 3; Figure 3a), and WOMAC™ Function subscale score at week 13 (Table 4; Figure 4a).

For each variable (OA pain, patient's global assessment of disease activity, and the WOMAC™ Function subscale score), each of the analysis methods showed statistically significant superiority of lumiracoxib 100 mg od (with or without initial dose) or celecoxib 200 mg od to placebo (Table 2). Moreover, the observed treatment effect (for example, the difference in the percentage of patients in the active treatment *minus *placebo group) was frequently above the 10% threshold, which is usually considered as reflecting a clinical relevance of the results. No statistical differences were observed between lumiracoxib 100 mg od (with or without initial dose) and celecoxib for any of the variables analysed.

For OA pain, the corresponding NNTs (95% confidence intervals [CIs]) for the active treatments at week 13 were 12.8 (7.97 to 32.80), 10.1 (6.84 to 19.65), and 14.9 (8.74 to 50.79) for lumiracoxib 100 mg od, lumiracoxib 100 mg od with initial dose, and celecoxib 200 mg od, respectively.

For patient's global assessment of disease activity, the corresponding NNTs (95% CIs) for the active treatment groups at week 13 were 9.0 (6.32 to 15.46), 8.2 (5.91 to 13.30), and 12.7 (8.00 to 31.27) for lumiracoxib 100 mg od, lumiracoxib 100 mg od with initial dose, and celecoxib 200 mg od, respectively.

For the WOMAC™ Function subscale score, the corresponding NNTs (95% CIs) for all active treatment groups at week 13 were 8.3 (6.00 to 13.52), 8.4 (6.05 to 13.79), and 10.8 (7.24 to 21.65) for lumiracoxib 100 mg od, lumiracoxib 100 mg od with initial dose, and celecoxib 200 mg od, respectively.

#### Early effect at 2 weeks

Significantly more patients were satisfied with treatment as defined by MCII or PASS in the lumiracoxib 100 mg od, lumiracoxib 100 mg od with initial dose, and celecoxib 200 mg od groups compared with those in the placebo group at week 2 (Tables 2, 3, 4, 5).

#### Achievement of sustained PASS during the study

Achievement of a sustained satisfactory symptom state according to PASS, maintained from the clinic visit during the study until the end of the study, is summarised in Figures 2b, 3b, and 4b for the three outcome measures, respectively. At all time points, all the performed analyses showed statistically significant differences in favour of lumiracoxib 100 mg od (with or without initial dose) or celecoxib 200 mg od over placebo.

#### Achievement of PASS for multiple variables

Table 5 shows the percentage of patients who achieved a PASS for one or more, two or more, and three variables at week 13. A high proportion of patients receiving lumiracoxib 100 mg od (57.2%), lumiracoxib 100 mg od with initial dose (59.4%), or celecoxib (54.5%) achieved a satisfactory symptom state according to PASS for at least one of the three variables evaluated.

#### Response by OMERACT-OARSI criteria

**Figure 5:**
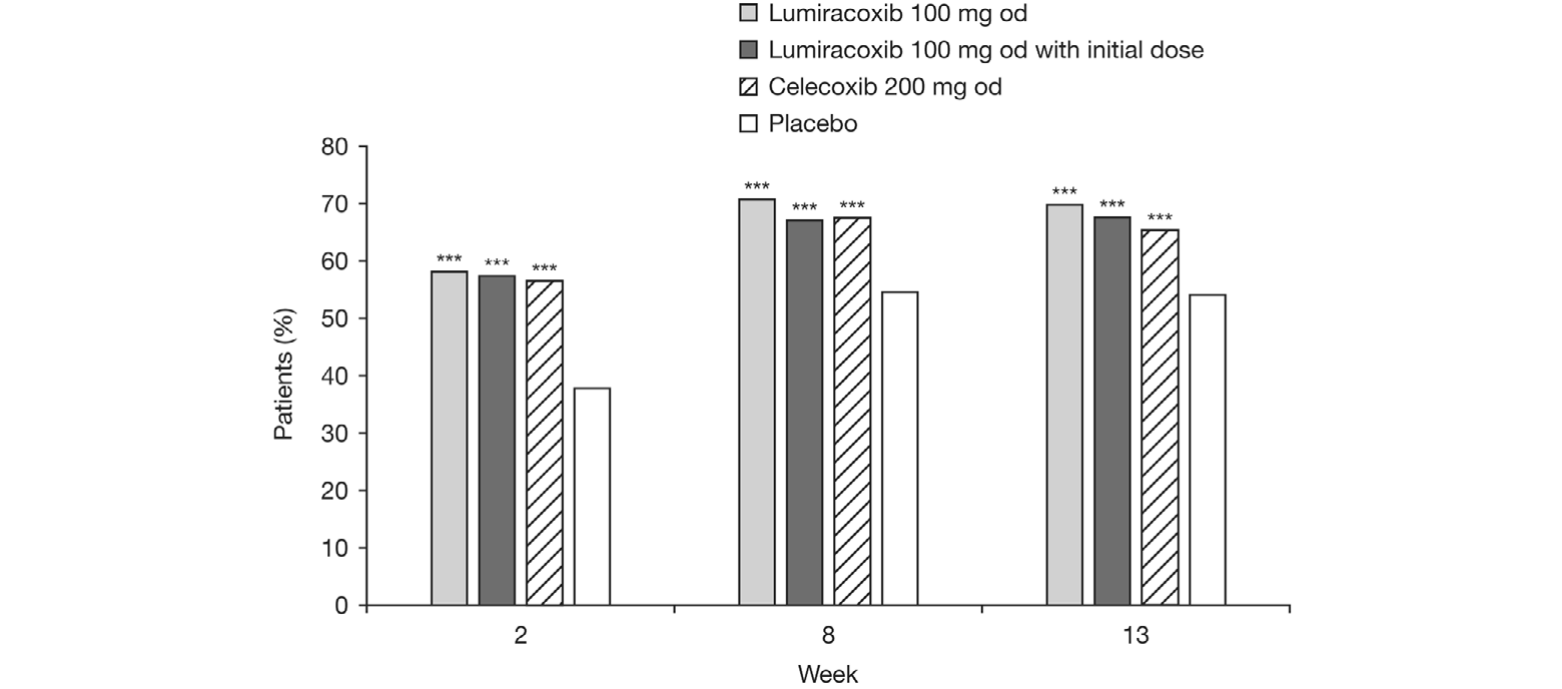
Response to treatment according to criteria of Outcome Measures in Clinical Trials-Osteoarthritis Research Society International. ***P < 0.001 versus placebo logistic regression model unadjusted for multiple comparisons.

At all time points, a significantly greater number of patients in the lumiracoxib 100 mg od, lumiracoxib 100 mg od with initial dose, and the celecoxib 200 mg od groups responded to treatment as defined by the OMERACT-OARSI criteria compared with placebo (Figure 5).

## Discussion

This study suggests that the analysis of clinical trials evaluating quick-acting symptomatic drugs, such as nonsteroidal anti-inflammatory drugs and selective COX-2 inhibitors, in OA can be adequately performed not only by using a conventional approach (for example, by comparing changes in conventional outcome variables by treatment group during the study) but also by referring to the novel concept of PASS.

The statistical analysis of continuous variables is usually considered as more powerful than the analysis of dichotomous variables. The concept of PASS does necessitate a switch from a continuous variable (for example, 0- to 100-mm VAS) to a dichotomous variable (for example, acceptable state yes/no). Despite such potential weakness, this study suggests that, when evaluating active drugs such as lumiracoxib or celecoxib versus placebo in OA, the results still reach statistical significance.

It is often difficult for clinicians to interpret clinical relevance of results obtained using conventional approaches. The presentation of the percentage of patients who consider themselves to have an acceptable symptom state is therefore of great relevance to clinicians and is a useful assessment variable in clinical trials. Finally, such presentation facilitates the evaluation of the reported placebo-controlled trials. In the case of symptomatic treatment of OA, a 10-point difference between the placebo group and the active group is usually considered as clinically relevant (expert's opinion). Two previous studies have shown that lumiracoxib 100 mg od (with or without initial dose) and celecoxib 200 mg od were significantly better than placebo for improving conventional measures of OA outcomes in clinical trials [6,7]. These data were reanalysed for PASS, and for all the evaluated variables, the observed treatment effect of lumiracoxib 100 mg od with or without initial dose or celecoxib 200 mg od over placebo was near or above the expected 10-point difference, suggesting that observed results are not only of statistical significance but also of clinical relevance. These results were also observed at 2 weeks, showing that this concept also allows evaluation of efficacy early in treatment.

A high proportion of patients in the active groups achieved an acceptable state according to PASS for at least one measure of symptom relief. Almost half of these patients achieved the PASS threshold for all three measures. Fewer patients achieved the PASS threshold than the MCII threshold. This differentiation between the two endpoints implies that PASS is an important new assessment measure in pain research.

## Conclusion

This investigation found that a high proportion of patients with OA of the knee, who were treated with lumiracoxib 100 mg od or celecoxib 200 mg od, reported an acceptable symptom state after 13 weeks for multiple measures. Studies of longer duration and/or in other musculoskeletal conditions and/or evaluating different treatment modalities are still required to further validate the PASS concept.

## Abbreviations

CI = confidence interval; COX-2 = cyclo-oxygenase-2; LK 3.1 = Likert version 3.1; MCII = Minimal Clinically Important Improvement; NNT = number needed to treat; OA = osteoarthritis; od = once daily; OMERACT-OARSI = Outcome Measures in Clinical Trials-Osteoarthritis Research Society International; PASS = Patient Acceptable Symptom State; VAS = visual-analogue scale; WOMAC™ = Western Ontario and McMaster Universities Osteoarthritis Index.

## Competing interests

AM and XG are employees of Novartis Pharma AG (Basel, Switzerland). SY is an employee of Novartis Pharmaceuticals Corporation (East Hanover, NJ, USA). MD has participated in different advisory boards evaluating coxibs such as rofecoxib, lumiracoxib, celecoxib, and etoricoxib; he has also participated as a speaker in different symposia organised by pharmaceutical companies in charge of the development and/or the marketing of coxibs (for example, Merck [Darmstadt, Germany], Pfizer Inc [New York, NY, USA], and Novartis Pharma AG [Basel, Switzerland]).

## Authors' contributions

MD, AM, SY, and XG conceived the study and participated in its design and coordination. SY and AM performed the statistical analysis. All authors contributed to drafting the manuscript. All authors read and approved the final manuscript.

## Figures and Tables

**Table 1:** Patient demographics and baseline disease characteristics

	Lumiracoxib 100 mg od (*n *= 811)	Lumiracoxib 100 mg od with initial dose (*n *= 805)	Celecoxib 200 mg od (*n *= 813)	Placebo (*n *= 806)
Age in years, mean ± SD	61.3 ± 10.6	61.9 ± 10.3	61.6 ± 10.6	61.3 ± 10.4
Females, *n *(%)	526 (64.9)	539 (67.0)	535 (65.8)	539 (66.9)
Body mass index in kg/m^2^, mean ± SD	31.2 ± 6.3	31.2 ± 6.1	31.1 ± 6.5	31.1 ± 6.2
Race, *n *(%)				
Caucasian	773 (95.3)	755 (93.8)	756 (93.0)	765 (94.9)
Black/African-American	19 (2.3)	20 (2.5)	30 (3.7)	19 (2.4)
Disease duration in years, mean ± SD	5.6 ± 6.8	5.6 ± 7.3	5.5 ± 6.9	5.4 ± 6.6
Baseline OA pain intensity (VAS [mm])	65.2 (13.8)	64.9 (13.7)	65.6 (13.8)	64.9 (13.2)
Baseline patient's global assessment of disease activity (VAS [mm])	63.1 (17.2)	62.7 (17.1)	62.3 (17.7)	62.7 (16.6)
Baseline WOMAC™ LK 3.1 Function score (VAS)	36.2 (10.8)	36.4 (11.3)	36.6 (10.9)	36.5 (10.7)

OA, osteoarthritis; od, once daily; SD, standard deviation; VAS, visual-analogue scale; WOMAC™ LK 3.1, Western Ontario and McMaster Universities Osteoarthritis Index Likert version 3.1.

**Table 2:** OA pain intensity

	Lumiracoxib 100 mg od (*n *= 811)	Lumiracoxib 100 mg od with initial dose (*n *= 805)	Celecoxib 200 mg od (*n *= 813)	Placebo (*n *= 806)
Mean change from baseline at week 2 ± SD^a^	-20.1^b ^± 21.97	-20.9^b ^± 22.50	-20.2^b ^± 21.86	-12.1 ± 19.92
Mean change from baseline at week 13 ± SD^a^	-26.0^b ^± 24.83	-26.0^b ^± 24.92	-25.4^b ^± 25.03	-19.8 ± 24.75
Response by MCII^a^				
Responders at week 2, *n *(%)	383 (47.2)	378 (47.0)	392 (48.3)	254 (31.6)
Odds ratio versus placebo^c ^(95% CI)	1.94^b ^(1.58–2.38)	1.92^b ^(1.57–2.35)	2.02^b ^(1.65–2.48)	NA
Odds ratio versus celecoxib^c ^(95% CI)	0.96^d ^(0.79–1.17)	0.95^d ^(0.78–1.15)	NA	NA
Responders at week 13, *n *(%)	484 (59.7)	489 (60.7)	463 (57.0)	393 (48.8)
Odds ratio versus placebo^c ^(95% CI)	1.55^b ^(1.27–1.89)	1.62^b ^(1.33–1.98)	1.39^b ^(1.14–1.69)	NA
Odds ratio versus celecoxib^c ^(95% CI)	1.12^d ^(0.92–1.36)	1.17^d ^(0.96–1.42)	NA	NA
Patients considering their current state as satisfactory by PASS^e^				
Satisfied patients at week 2, *n *(%)	251 (30.9)	270 (33.5)	242 (29.8)	137 (17.0)
Odds ratio versus placebo^c ^(95% CI)	2.19^b ^(1.73–2.77)	2.46^b ^(1.95–3.12)	2.07^b ^(1.63–2.62)	NA
Odds ratio versus celecoxib^c ^(95% CI)	1.06^d ^(0.86–1.31)	1.19^d ^(0.97–1.47)	NA	NA
Satisfied patients at week 13, *n *(%)	351 (43.3)	365 (45.3)	343 (42.2)	286 (35.5)
Odds ratio versus placebo^c ^(95% CI)	1.39^b ^(1.14–1.70)	1.51^b ^(1.23–1.84)	1.33^f ^(1.09–1.62)	NA
Odds ratio versus celecoxib^c ^(95% CI)	1.05^d ^(0.86–1.27)	1.14^d ^(0.93–1.38)	NA	NA

^a^A patient was considered a responder by MCII if his/her change from baseline for OA pain intensity was decreased by greater than or equal to 19.9 mm. ^b^*p *< 0.001 versus placebo. ^c^Multiple logistic regression model with treatment as main effect. Pairwise comparisons were tested using two-sided significance unadjusted for multiple comparisons. ^d^*p *value non-significant. ^e^A patient was considered as achieving a satisfactory state according to PASS if his/her value for OA pain intensity was less than or equal to 32.3 mm. ^f^*p *< 0.01 versus placebo. CI, confidence interval; MCII, Minimal Clinically Important Improvement; NA, not applicable; OA, osteoarthritis; od, once daily; PASS, Patient Acceptable Symptom State; SD, standard deviation.

**Table 3:** Patient's global assessment of disease activity

	Lumiracoxib 100 mg od (*n *= 811)	Lumiracoxib 100 mg od with initial dose (*n *= 805)	Celecoxib 200 mg od (*n *= 813)	Placebo (*n *= 806)
Mean change from baseline at week 2 ± SD^a^	-18.4^b ^± 23.62	-19.0^b ^± 23.83	-17.1^b ^± 23.72	-9.5 ± 20.81
Mean change from baseline at week 13 ± SD^a^	-24.2^b ^± 25.83	-23.2^b ^± 25.57	-21.3^b ^± 26.78	-16.3^b ^± 25.19
Response by MCII^c ^at week 13				
Responders at week 2, *n *(%)	359 (44.3)	369 (45.8)	352 (43.3)	242 (30.1)
Odds ratio versus placebo^d ^(95% CI)	1.85^b ^(1.51–2.27)	1.97^b ^(1.60–2.42)	1.78^b ^(1.45–2.18)	NA
Odds ratio versus celecoxib^d ^(95% CI)	1.04^e ^(0.85–1.27)	1.11^e ^(0.91–1.35)	NA	NA
Responders at week 13, *n *(%)	465 (57.3)	456 (56.6)	432 (53.1)	357 (44.3)
Odds ratio versus placebo^d ^(95% CI)	1.69^b ^(1.39–2.05)	1.64^b ^(1.35–2.00)	1.42^b ^(1.17–1.73)	NA
Odds ratio versus celecoxib^d ^(95% CI)	1.19^e ^(0.97–1.44)	1.15^e ^(0.95–1.40)	NA	NA
Satisfaction with treatment by PASS^f^				
Satisfied patients at week 2, *n *(%)	240 (29.6)	255 (31.7)	234 (28.8)	140 (17.4)
Odds ratio versus placebo^d ^(95% CI)	2.00^b ^(1.58–2.53)	2.21^b ^(1.74–2.79)	1.92^b ^(1.52–2.44)	NA
Odds ratio versus celecoxib^d ^(95% CI)	1.04^e ^(0.84–1.29)	1.15^e ^(0.93–1.42)	NA	NA
Satisfied patients at week 13, *n *(%)	347 (42.8)	353 (43.9)	321(39.5)	255 (31.6)
Odds ratio versus placebo^d ^(95% CI)	1.62^b ^(1.32–1.98)	1.69^b ^(1.38–2.07)	1.41^b ^(1.15–1.73)	NA
Odds ratio versus celecoxib^d ^(95% CI)	1.15^e ^(0.94–1.40)	1.20^e ^(0.98–1.46)	NA	NA

^a^*p *values for comparison with placebo in analysis of covariance adjusting for study and baseline. ^b^*p *< 0.001 versus placebo. ^c^A patient was considered a responder by MCII if his/her change from baseline for patient's global assessment was decreased by greater than or equal to 18.3 mm. ^d^Multiple logistic regression model with treatment as main effect. Pairwise comparisons were tested using two-sided significance unadjusted for multiple comparisons. ^e^*p *value non-significant. ^f^A patient was considered as achieving a satisfactory state according to PASS if his/her value for patient's global assessment was less than or equal to 32.0 mm. CI, confidence interval; MCII, Minimal Clinically Important Improvement; NA, not applicable; od, once daily; PASS, Patient Acceptable Symptom State; SD, standard deviation.

**Table 4:** WOMAC™ LK 3.1 Function subscale score response

	Lumiracoxib 100 mg od (*n *= 811)	100 mg od with initial dose (*n *= 805)	Celecoxib 200 mg od (*n *= 813)	Placebo (*n *= 806)
Mean change from baseline at week 2 ± SD^a^	-8.7^b ^± 10.71	-8.9^b ^± 11.19	-8.7^b ^± 10.61	-4.1 ± 9.41
Mean change from baseline at week 13 ± SD^a^	-11.2^b ^± 12.65	-11.2^b ^± 12.71	-10.5^b ^± 12.41	-7.2 ± 12.62
Response by MCII^c^				
Responders at week 2, *n *(%)	425 (52.7)	413 (51.7)	422 (52.1)	264 (32.9)
Odds ratio versus placebo^d ^(95% CI)	2.27^b ^(1.86–2.78)	2.18^b ^(1.78–2.67)	2.22^b ^(1.81–2.72)	NA
Odds ratio versus celecoxib^d ^(95% CI)	1.02^e ^(0.84–1.24)	0.98^e ^(0.81–1.20)	NA	NA
Responders at week 13, *n *(%)	506 (62.7)	495 (62.0)	490 (60.5)	378 (47.1)
Odds ratio versus placebo^d ^(95% CI)	1.89^b ^(1.55–2.31)	1.83^b ^(1.50–2.23)	1.72^b ^(1.41–2.10)	NA
Odds ratio versus celecoxib^d ^(95% CI)	1.10^e ^(0.90–1.34)	1.06^e ^(0.87–1.30)	NA	NA
Patients considering their current state as satisfactory by PASS^f^				
Satisfied patients at week 2, *n *(%)	262 (32.3)	271 (33.7)	259 (31.9)	154 (19.1)
Odds ratio versus placebo^d ^(95% CI)	2.02^b ^(1.61–2.54)	2.15^b ^(1.71–2.71)	1.98^b ^(1.57–2.49)	NA
Odds ratio versus celecoxib^d ^(95% CI)	1.02^e ^(0.83–1.26)	1.09^e ^(0.88–1.34)	NA	NA
Satisfied patients at week 13, *n *(%)	337 (41.6)	333 (41.4)	315 (38.7)	238 (29.5)
Odds ratio versus placebo^d^	1.70^b ^(1.38–2.09)	1.69^b ^(1.37–2.07)	1.51^b ^(1.23–1.86)	NA
Odds ratio versus celecoxib^d^	1.12^e ^(0.92–1.37)	1.12^e ^(0.92–1.36)	NA	NA

^a^*p *values for comparison with placebo in analysis of covariance adjusting for study and baseline. ^b^*p *< 0.001 versus placebo. ^c^A patient was considered a responder by MCII if his/her change from baseline for WOMAC™ LK 3.1 Function was decreased by greater than or equal to 6.19 (converted from VAS). ^d^Multiple logistic regression model with treatment as main effect. Pairwise comparisons were tested using two-sided significance unadjusted for multiple comparisons. ^e^*p *value non-significant. ^f^A patient was considered as achieving a satisfactory state according to PASS if his/her value for WOMAC™ LK 3.1 Function was less than or equal to 21.08 (converted from VAS). CI, confidence interval; MCII, Minimal Clinically Important Improvement; NA, not applicable; od, once daily; PASS, Patient Acceptable Symptom State; SD, standard deviation; VAS, visual-analogue scale; WOMAC™ LK 3.1, Western Ontario and McMaster Universities Osteoarthritis Index Likert version 3.1.

**Table 5:** Patients achieving a satisfactory symptom state according to one or more, two or more, or three PASS criteria^a^

	Lumiracoxib 100 mg od (*n *= 811)	Lumiracoxib 100 mg od with initial dose (*n *= 805)	Celecoxib 200 mg od (*n *= 813)	Placebo (*n *= 806)
Patients considering their current state as satisfactory at week 2 according to:				
One or more PASS criteria, *n *(%)	396 (48.8)	393 (48.8)	367 (45.1)	241 (29.9)
Two or more PASS criteria, *n *(%)	229 (28.2)	259 (32.2)	226 (27.8)	128 (15.9)
Three PASS criteria, *n *(%)	128 (15.8)	144 (17.9)	142 (17.5)	62 (7.7)
Patients considering their current state as satisfactory at week 13 according to:				
One or more PASS criteria, *n *(%)	464 (57.2)	478 (59.4)	443 (54.5)	367 (45.5)
Two or more PASS criteria, *n *(%)	340 (41.9)	343 (42.6)	325 (40.0)	256 (31.8)
Three PASS criteria, *n *(%)	231 (28.5)	230 (28.6)	211 (26.0)	156 (19.4)

^a^Osteoarthritis pain, patient's global assessment of disease activity, or WOMAC™ LK 3.1 Function subscale score. od, once daily; PASS, Patient Acceptable Symptom State; WOMAC™ LK 3.1, Western Ontario and McMaster Universities Osteoarthritis Index Likert version 3.1.
